# Low hepatitis C prevalence in Belgium: implications for treatment reimbursement and scale up

**DOI:** 10.1186/s12889-018-6347-z

**Published:** 2019-01-08

**Authors:** Amber Litzroth, Vanessa Suin, Chloé Wyndham-Thomas, Sophie Quoilin, Gaëtan Muyldermans, Thomas Vanwolleghem, Benoît Kabamba-Mukadi, Vera Verburgh, Marjorie Jacques, Steven Van Gucht, Veronik Hutse

**Affiliations:** 1 Scientific directorate Epidemiology and public health, Sciensano, Juliette Wytsmanstreet 14, 1050 Brussels, Belgium; 2 Scientific directorate Infectious diseases in humans, Sciensano, Juliette Wytsmanstreet 14, 1050 Brussels, Belgium; 3 Laboratory of Experimental Medicine and Pediatrics, Faculty of Medicine and Health Sciences, University of Antwerp, Campus Drie Eiken Building S, Universiteitsplein 1, 2610 Wilrijk, Belgium; 4 Department of Gastroenterology and Hepatology, Antwerp University Hospital, Wilrijkstraat 10, 2650 Edegem, Belgium; 5 Microbiology department, Cliniques universitaires Saint-Luc, Université Catholique de Louvain (UCL), Avenue Hippocrate 10, 1200 Brussels, Belgium

**Keywords:** Hepatitis C, Prevalence, Chronic hepatitis C virus infection, Seroprevalence, Direct-acting antivirals, Belgium

## Abstract

**Background:**

Prevalence data of chronic hepatitis C virus (HCV) infection are needed to estimate the budgetary impact of reimbursement of direct-acting antivirals (DAAs). In Belgium, the restricted reimbursement criteria are mainly guided by regional seroprevalence estimates of 0.87% from 1993 to 1994. In this first Belgian nationwide HCV prevalence study, we set out to update the seroprevalence and prevalence of chronic HCV infection estimates in the Belgian general population in order to guide decisions on DAA reimbursement.

**Methods:**

Residual sera were collected through clinical laboratories. We collected data on age, sex and district. HCV antibody status was determined with ELISA and confirmed with a line-immunoassay (LIA). In specimens with undetermined or positive LIA result, HCV viral load was measured. Specimens were classified seronegative, seropositive with resolved infection, indicative of chronic infection and with undetermined HCV status according to the test outcomes. Results were standardized for age, sex and population per district, and adjusted for clustered sampling.

**Results:**

In total 3209 specimens, collected by 28 laboratories, were tested. HCV seropositivity in the Belgian general population was estimated to be 0.22% (95% CI: 0.09–0.54%), and prevalence of chronic HCV infection 0.12% (95% CI: 0.03–0.41). In individuals of 20 years and older, these estimates were 0.26% (95% CI: 0.10–0.64%) and 0.13% (95% CI: 0.04–0.43), respectively. Of the total estimated number of HCV seropositive individuals in Belgium, 66% were between 50 and 69 years old.

**Conclusions:**

Prevalence of HCV seropositivity and chronic infection in the Belgian general population were low and comparable to many surrounding countries. These adjusted prevalences can help estimate the cost of reimbursement of DAAs and invite Belgian policy makers to accelerate the scaling up of reimbursement, giving all chronically infected HCV patients a more timely access to treatment.

## Background

The newly developed direct-acting antivirals (DAAs) provoked a revolution in the treatment of chronic hepatitis C virus (HCV) infection. With a 95% success rate, minimal side effects and shorter treatment duration, the burden of the disease can drastically be reduced. Elimination of hepatitis C could now be within reach [1, 2] and scaling up of treatment of chronic HCV infection is crucial in the recently set hepatitis C elimination targets for 2030 of the World Health Organization (WHO) [3, 4]. Several studies have indicated that treatment of all those with chronic HCV infection is cost-effective [5–7]. However, cost-effectiveness does not imply affordability and the high cost of DAAs has resulted in reimbursement restrictions in many countries. Reimbursement criteria often target more severe liver fibrosis stages and sometimes include restrictions concerning drugs or alcohol use [8, 9]. In Western Europe, Belgium remains one of the few countries restricting DAA reimbursement to fibrosis stage F2 or higher [9].

In order to estimate the cost of different reimbursement strategies, data on the prevalence of chronic HCV infection are needed. In Belgium, no nationwide HCV prevalence data exist and the last regional study on serum samples was conducted in 1993–1994 without distinction between resolved and current infection [10, 11]. The Belgian restricted DAA reimbursement criteria are therefore mainly guided by the 1993–1994 seroprevalence estimate of 0.87%, the lack of recent prevalence data and caution concerning the budgetary impact of different reimbursement strategies.

Here, we set out to update the HCV seroprevalence and prevalence of chronic HCV infection estimates in the Belgian general population in order to guide further decisions on DAA reimbursement criteria.

## Methods

### Study design, specimen and data collection and ethics

In the context of a multiple disease seroprevalence study, residual sera were collected through clinical laboratories between July 2013 and January 2015. The sample size per age group aimed for was based on estimations of the European Sero-Epidemiology Network (ESEN), which included an oversampling of individuals < 20 years old [12]. This strategy was chosen because the serum bank would also be tested for a number of other diseases, among which some vaccine preventable diseases for which we wanted to obtain precise seroprevalence estimates in the younger age groups.

In order not to oversample an ill or susceptible population, laboratories were asked to preferably collect specimens from surgery, orthopaedic, emergency and otorhinolaryngology wards. We also asked the laboratories to exclude specimens from immunocompromised patients, patients from intensive care wards and with evidence of multiple blood transfusions. Laboratories were not asked to systematically retrieve this information, but to only exclude these specimens if the information was known to the person responsible for the collection.

We collected data on age, sex and district. Participating laboratories were retrospectively asked if they performed analyses for correctional facilities and/or needle exchange programs. The protocol was approved by the Ethics committee of the Antwerp University Hospital and the University of Antwerp.

### Laboratory testing strategy and specimen classification

Laboratory tests were performed in 2017–2018 by the National reference center for hepatitis C. HCV antibody status was determined using the HCV 4.0 Antibody Enzygnost ELISA (Siemens healthcare diagnostics, Munich, Germany). ELISA anti-HCV antibody-positive specimens were confirmed with a line-immunoassay (LIA), the INNO-LIA HCV Score (Fujirebio, Gent, Belgium). In specimens with undetermined or positive LIA result, HCV viral load was measured by reverse transcriptase real-time PCR (RT-qPCR) with the Abbott RealTime HCV kit on the m2000 system (Abbott, Illinois, USA).

Specimens were classified seropositive for HCV if they were either LIA-positive, or had an undetermined LIA result with positive PCR. PCR-positive specimens were considered indicative of chronic HCV infection, while PCR-negative LIA-positive specimens were considered to indicate a resolved infection. PCR-negative specimens with an undetermined LIA result were considered to have an undetermined HCV status. All other specimens were classified HCV-seronegative.

### Statistical analysis

We calculated prevalence of HCV seropositivity and chronic infection and the 95% confidence intervals (95% CI) in the general Belgian population, per age group (younger than 20 as one group and further by decade), by region and by sex. A weighting factor for age, population per district and sex was applied to account for underrepresentation of certain groups. The weights applied were the inverse of the sampling fraction. To calculate the sampling fraction we used Belgian population data of 2013, made available by the Belgian statistical office Statbel. We adjusted for clustered sampling by defining laboratories as the primary sampling units. For subgroups with zero positive observations, we calculated the 95% CI with the binomial approach. Based on the estimated prevalences, we calculated the estimated number of HCV seropositive and chronically infected individuals in the different demographic groups. We estimated the clearance rate by calculating the percentage of estimated number of seropositive non-viraemic individuals among the estimated number of seropositive individuals in the Belgian population. We used Stata version 14.0 (Stata Corporation, Texas, US) for all statistical analyses.

## Results

In total 3209 specimens, collected by 28 laboratories, were tested. Specimens were representative of the Belgian population in terms of sex and fairly representative for population per region, although the Walloon region was slightly undersampled. Individuals under 20 years of age were oversampled, as aimed for in the ESEN sampling strategy (Table 1). Of the 22 laboratories that shared this information, nine reported to receive samples from correctional facilities, and one from needle exchange programs.

**Table 1:** Demographic characteristics of the study sample and the general population in 2013 in Belgium

Demographic characteristic		Study population *n* = 3209		Total Belgian population 2013 *n* = 11,099,554	
		*n*	%	*n*	%
Sex	Female	1607	50.1	5,652,066	50.9
	Male	1602	49.9	5,447,488	49.1
Region	Brussels-Capital	372	11.6	1,154,635	10.4
	Flanders	2044	63.7	6,381,859	57.5
	Wallonia	793	24.7	3,563,060	32.1
Age group (in years)	< 20	1735	54.1	2,516,257	22.7
	20–29	375	11.7	1,395,074	12.6
	30–39	363	11.3	1,451,565	13.1
	40–49	188	5.9	1,600,664	14.4
	50–59	193	6.0	1,525,220	13.7
	60–69	176	5.5	1,206,822	10.9
	70–79	80	2.5	816,895	7.4
	≥80	99	3.1	587,057	5.3

**Fig. 1:**
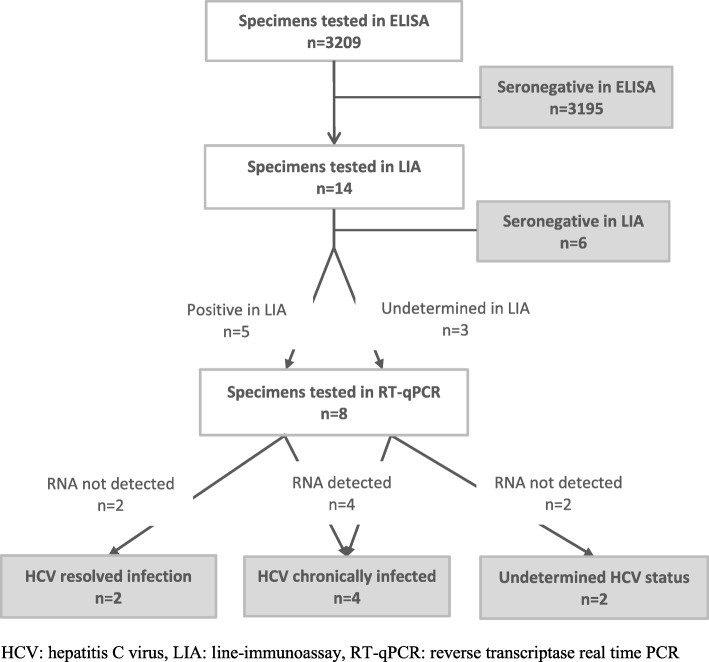
HCV testing strategy and results, 2013–2014 seroprevalence study, Belgium

Of all specimens, 14 (0.44%) tested ELISA-positive and were further tested in LIA. Six of 14 (43%) were found to be seronegative. Of the eight samples tested in RT-qPCR, two were found seropositive with resolved infection, four were found to be indicative of chronic HCV infection, and of two the HCV status remained undetermined (Fig. 1). The seropositive samples were found in a 12, 34, 40, 50, 53 and 67 year old. The two latter had a resolved infection.

After weighting for sex, age and population per district and adjusting for clustered sampling, HCV seropositivity was estimated to be 0.22% (95% CI: 0.09–0.54%), and prevalence of chronic HCV infection 0.12% (95% CI: 0.03–0.41%). In those of 20 years and older, these estimates were 0.26 (95% CI: 0.10–0.64) and 0.13 (95% CI: 0.04–0.43), respectively. No statistically significant differences were detected by sex, region or age group. We estimated there to be 24,420 (95% CI: 9990–59,940) HCV seropositive and 13,320 (95% CI: 3770–45,510) chronically infected individuals in the Belgian general population. The estimated clearance rate was 45%. Of the total estimated number of HCV seropositive individuals in Belgium, 66% were between 50 and 69 years old (Table 2, Table 3).

**Table 2:** Estimated hepatitis C virus seroprevalence and estimated number of seropositive individuals, 2013–2014, Belgium

Demographic characteristic		HCV seropositivity 2013–2014				
		Number in study sample	Prevalence in Belgium		Individuals in Belgium	
			%	95% CI	*n*	95% CI
Sex	Female	2	0.13	0.03–0.59	7200	1510–33,520
	Male	4	0.32	0.1–1.03	17,450	5370–56,380
Region	Brussels-Capital	2	1.05	0.35–3.15	12,180	4020–36,320
	Flanders	4	0.21	0.07–0.66	13,300	4180–42,180
	Wallonia	0	0	0–0.46	0	0–16,390
Age group (in years)	< 20	1	0.08	0.01–0.54	1990	290–13,700
	≥20	5	0.26	0.10–0.64	22,220	8890–55,420
	20–29	0	0	0–0.98	0	0–13,670
	30–39	1	0.31	0.04–2.27	4490	600–32,890
	40–49	1	0.13	0.02–1.14	2150	250–18,230
	50–59	2	0.74	0.16–3.41	11,240	2380–52,030
	60–69	1	0.44	0.06–3.34	5290	680–40,260
	70–79	0	0	0–4.51	0	0–36,840
	≥80	0	0	0–3.66	0	0–21,490
All		6	0.22	0.09–0.54	24,420	9990–59,940

Legend: *HCV*: hepatitis C virus, *CI*: confidence interval

**Table 3:** Estimated prevalence of chronic HCV infection and estimated number of affected individuals, 2013–2014, Belgium

Demographic characteristic		HCV chronic infection 2013–2014				
		Number in study sample	Prevalence in Belgium		Individuals in Belgium	
			%	95% CI	*n*	95% CI
Sex	Female	1	0.04	0.005–0.34	2280	280–18,950
	Male	3	0.20	0.05–0.85	10,770	2500–46,130
Region	Brussels-Capital	0	0	0–0.99	0	0–11,430
	Flanders	4	0.21	0.07–0.66	13,300	4130–42,180
	Wallonia	0	0	0–0.46	0	0–16,390
Age group (in years)	< 20	1	0.08	0.01–0.54	1990	290–13,700
	≥20	3	0.13	0.04–0.43	10,900	3220–36,790
	20–29	0	0	0–0.98	0	0–13,670
	30–39	1	0.31	0.04–2.27	4490	600–32,890
	40–49	1	0.13	0.02–1.14	2150	250–18,230
	50–59	1	0.27	0.03–2.3	4180	490–35,130
	60–69	0	0	0–2.07	0	0–24,980
	70–79	0	0	0–4.51	0	0–36,840
	≥80	0	0	0–3.66	0	0–21,480
All		4	0.12	0.03–0.41	13,320	3770–45,510

Legend: *HCV*: hepatitis C virus, *CI*: confidence interval

## Discussion

In this first Belgian nationwide HCV prevalence study, we estimated the HCV seroprevalence to be 0.22% which is considerably lower than the 0.87% found in the Flanders region in 1993–1994 by Beutels et al. [10]. The exact reasons for this difference are difficult to determine. We believe that it reflects, at least partially, a true decrease in HCV prevalence, due to both a decline in incidence and a natural turnover of HCV infected individuals, as the interval between the collection of both serum banks was exactly 20 years. The decline in incidence is achieved through efforts made to eliminate nosocomial transmission by screening of blood donors [13] and haemophilia patients, through programs such as needle exchanges programs targeting IDU, and through treatment breakthroughs in the last decades. Previously, Jadoul et al. reported a two-fold decrease in HCV prevalence between 1991 and 2000 in Belgian hemodialysis patients [14], hereby showing a considerable impact of precautionary measures on the HCV prevalence in certain risk groups. However, we acknowledge that differences in study design may have contributed as well to the different estimations. Although Beutels et al. also used residual sera and sampled similar hospital wards, they slightly oversampled migrants, without correcting for this afterwards, and did not ask for exclusion of known immunocompromised patients or patients with evidence of multiple blood transfusions. A different representation of these populations in both studies may have contributed to the different estimates.

On the other hand, our estimate of HCV seroprevalence is slightly higher than the 0.12% found in a saliva-based study in the Flanders region in 2003 [11]. In 2003, participants were recruited into the study and saliva tests, with a slightly lower sensitivity than serum tests [11], were used to test for the presence of HCV antibodies. Both these factors could explain our, slightly higher, estimate.

Within Europe, nationwide seroprevalence estimates range from 0.22 to 3.5%, with countries in Southern and Eastern Europe generally being the most affected [15–17]. Our estimate is comparable to the 2012 Dutch serum bank-based estimate of 0.3% [16], and is exactly the same as the Dutch model-based estimate of 0.22% in individuals between 15 and 79 years of age [17]. The first of these studies recruited participants into the study, which could lead to an underestimation, however, the second one used a model to account for underrepresentation of specific risk groups and estimated the seroprevalence to be slightly lower.

The estimated Belgian prevalence of chronic HCV infection of 0.12% found in this study is substantially lower than the 2015 model-based viraemic prevalence estimate of 0.6% [18], which is predominantly driven by the seroprevalence estimate of 1993–1994. Our updated estimate is comparable to the Dutch model-based viraemic prevalence estimate of 0.1% and slightly lower than the 0.3% estimates of other surrounding countries like the UK, France and Germany [18]. However, some countries in the region, such as Ireland and Luxemburg, still report higher viraemic prevalences, with 0.6 and 0.9% respectively [18], this could be due to different population demographics and a different contribution of risk groups, such as IDU, to the estimates.

Our clearance rate of 45%, although based on a small number of positive samples, is higher than has been described by Seeff [19]. A similar clearance was observed by Garvey et al. in Ireland [20]. Unfortunately, we do not have details on treatment history of the participants, nor on the number of treated individuals in Belgium, but most likely some of these individuals were known HCV positive and have received treatment.

According to our study, 66% of HCV seropositive individuals in Belgium belong to the so called baby boom generation (born between 1945 and 1965). Due to the facts that this is based on a low number of seropositive individuals, that confidence intervals are wide and that information on risk factors is lacking, additional studies are needed to inform screening strategies. Even more so since screening the baby boom generation has proven to be effective in some countries, such as the United States [21], but other countries such as Germany [22] and the Netherlands [23], with population demographics more comparable to the Belgian one, have not found evidence to support this strategy.

In Belgium, reimbursement of DAAs is being gradually scaled up. Since January 2017, HCV patients with liver fibrosis stage F2 and higher and certain risk groups such as individuals with co-infection with HIV or hepatitis B virus, with blood clotting disorders, hemoglobinopathy, or with significant extrahepatic illness due to hepatitis C, dialysis patients and people who received an organ transplant hematopoietic stem cell transplant or bone marrow transplant (or are on the waiting list) [24], are considered eligible for treatment and reimbursement. However, neighbouring countries do not apply restrictions based on fibrosis stage, a strategy that is partly inspired by their low HCV viraemic prevalence [9, 18]. A 2016 Belgian economic analysis proposed a gradual scaling up of reimbursement of DAAs in order to limit the sudden budgetary impact [25]. The exact impact could not be calculated due to the lack of recent prevalence data of chronic HCV and lack of information on those infected. These new estimates would therefore call for a revision of the Belgian DAA reimbursement guidelines.

One of the main limitations of this study is that we may have underrepresented some specific risk groups, such as people with HIV/HCV coinfection, people with evidence of multiple transfusions, prisoners, migrants and injecting drug users (IDU). Due to exclusion of known immunocompromised individuals, we may have missed those people with HIV/HCV coinfection, which mainly occurs in men who have sex with men (MSM) and IDU. In 2013 there were 5650 diagnosed HIV-positive diagnosed MSM in Belgium [26]. Currently, no Belgian data on HCV seroprevalence in this group are available, but a meta-analysis estimated this to be 8.1% [27]. This would result in 458 HIV/HCV coinfected MSM in Belgium in 2013. In the same year, the total number of diagnosed HIV-positive IDU in Belgium was less than 500 [26]. These numbers show that even a complete exclusion of these groups would have had little impact on the estimates we provided. Unfortunately, the possible impact of excluding people with evidence of multiple transfusions cannot be calculated due to a lack of data. It could be argued that this is an important risk group that we have missed. Nevertheless, since the group was not systematically excluded as the information was not actively retrieved, the impact on our estimates was likely to be limited.

Although other high risk groups like prisoners, migrants and IDUs were not specifically excluded from the study, it is impossible to determine the extent to which they were representatively included in the study sample based on the data we had available. Almost half of the labs reported receiving samples from correctional facilities, but the access to public health care of prisoners is likely to be more restricted than that of the general population. Also IDU and certain migrant populations are less likely to access regular health care. Therefore, we have to acknowledge that our overall estimates might be biased towards medical care seekers and may represent a slight underestimation of the true prevalence. The use of emergency room samples, on the other hand, may have caused an overestimation, as higher prevalences in this population have been observed [28, 29].

Additionally, due to the oversampling of younger age groups, overall estimates and estimates in adults are less precise than they would have been with a population based sampling. For this reason, we had a low power to detect significant differences between age groups, sex or regions.

Finally, we cannot exclude an acute infection was misclassified as chronic which would result in an overestimation of the prevalence of chronicity. This does however not interfere with the conclusions of low seroprevalence and low prevalence of chronicity in the Belgian general population.

## Conclusion

In conclusion, prevalence of HCV seropositivity and chronic infection in the Belgian general population is low and comparable to many surrounding countries. The adjusted prevalence can help estimate the cost of reimbursement of DAAs and invites Belgian policy makers to accelerate the scaling up of reimbursement, giving all chronically infected HCV patients a more timely access to treatment. Prompt treatment will not only prevent or limit the liver damage, which paradoxically now has to occur before being considered eligible for treatment reimbursement, but will also stop further transmission, which is key to the elimination of hepatitis C. Further studies are however needed to estimate prevalence in risk groups and to inform a screening strategy.

## Abbreviations

CI: Confidence interval
DAA: Direct-acting antivirals
ESEN: European Sero-Epidemiology Network
HCV: Hepatitis C virus
IDU: Injecting drug users
LIA: Line-immunoassay
MSM: Men who have sex with men
RT-qPCR: Reverse transcriptase real-time PCR
WHO: World Health Organisation
